# An IHS-Based Pan-Sharpening Method for Spectral Fidelity Improvement Using Ripplet Transform and Compressed Sensing

**DOI:** 10.3390/s18113624

**Published:** 2018-10-25

**Authors:** Chen Yang, Qingming Zhan, Huimin Liu, Ruiqi Ma

**Affiliations:** 1 School of Urban Design, Wuhan University, 8 Donghu South Road, Wuhan 430072, China; yangchenwhu@whu.edu.cn (C.Y.); hmliu@whu.edu.cn (H.L.); 2 Collaborative Innovation Center of Geospatial Technology, 129 Luoyu Road, Wuhan 430079, China; 3 State Key Laboratory of Information Engineering in Surveying, Mapping and Remote Sensing, Wuhan University, Wuhan 430079, China; mrq_rs@163.com

**Keywords:** remote sensing, image fusion, ripplet transform, intensity-hue-saturation transform, sparse representation

## Abstract

Pan-sharpening aims at integrating spectral information from a multi-spectral (MS) image and spatial information from a panchromatic (PAN) image in a fused image with both high spectral and spatial resolutions. Numerous pan-sharpening methods are based on intensity-hue-saturation (IHS) transform, which may cause evident spectral distortion. To address this problem, an IHS-based pan-sharpening method using ripplet transform and compressed sensing is proposed. Firstly, the IHS transform is applied to the MS image to separate intensity components. Secondly, discrete ripplet transform (DRT) is implemented on the intensity component and the PAN image to obtain multi-scale sub-images. High-frequency sub-images are fused by a local variance algorithm and, for low-frequency sub-images, compressed sensing is introduced for the reconstruction of the intensity component so as to integrate the local information from both the intensity component and the PAN image. The specific fusion rule is defined by local difference. Finally, the inverse ripplet transform and inverse IHS transform are coupled to generate the pan-sharpened image. The proposed method is compared with five state-of-the-art pan-sharpening methods and also the Gram-Schmidt (GS) method through visual and quantitative analysis of WorldView-2, Pleiades and Triplesat datasets. The experimental results reveal that the proposed method achieves relatively higher spatial resolution and more desirable spectral fidelity.

## 1. Introduction

Remote sensing (RS) is a general approach for the knowledge extraction of the Earth’s surface structure and content through acquiring and interpreting the spectral characteristics from a great distance [1]. However, the well-known trade-off between spatial resolution and spectral resolution has always precluded the further application of RS products. Pan-sharpening is a desirable solution to settle such dilemma. In fact, most of the Earth observation satellite images, such as IKONOS, QuickBird, and the WorldView family (including WorldView-2/3/4), can only provide a panchromatic (PAN) image with high spatial resolution but low spectral resolution together, with a multi-spectral (MS) image with low spatial resolution but high spectral resolution, respectively [2]. Pan-sharpening, a special case of image fusion, is capable of obtaining an image with both high spatial and high spectral resolutions. In other words, the technique provides a fused image with the same spectral response as the MS image and with the spatial resolution of the PAN image. The pan-sharpened images have been proved to possess great potential for multiple applications, including land use/land cover (LULC) classification, land change detection and environment monitoring [3,4,5,6,7,8].

Generally, pan-sharpening methods can be classified into three main categories: component substitution (CS)-based methods, multi-resolution analysis (MRA)-based methods, and model-based methods. In addition, there are a number of subcategories of pan-sharpening methods. Detailed information regarding these subcategories has been investigated in the references [1,9,10,11]. Specifically, CS-based methods include intensity-hue-saturation (IHS) [12,13,14], the Gram-Schmidt (GS) method [1,15], and principle component analysis (PCA) [16,17]. CS-based methods firstly separate components into other color space by spectral transform (such as IHS, GS, and PCA). Secondly, the component with sufficient spatial details is substituted by the PAN image to enhance spatial resolution. Finally, the pan-sharpened MS images are obtained by performing an inverse spectral transform. The second group (MRA-based methods) usually applies digital filters, covering wavelet transform [18,19], contourlet transform [20], curvelet transform [21], Laplacian pyramids [22] and ripplet transform [23] to obtain the multi-scale representation of the MS images. The MRA-based approaches [24] firstly employ MRA transforms to decompose PAN images into multi-scale components. Then, the equivalent spatial information contained in the high-frequency component is injected into an up-sampled MS image. Detailed illustrations of CS- and MRA-based methods are well documented [24]. In addition, the edge-preserving filters are frequently integrated with MRA transforms [25]. The third category is model-based methods, such as compressed sensing-based methods [26,27] and sparse matrix factorization technique-based methods [28]. The compressed sensing-based methods perform pan-sharpening by reconstructing high-resolution MS images using image patches extracted from both source PAN and MS images. Each category of pan-sharpening method possesses inherent superiorities and limitations. CS-based methods, especially the IHS method, can improve the spatial resolution of MS images significantly while preserving high convenience and efficiency for implementation. However, CS-based methods result in spectral distortion in the substitution of components due to the ignorance of local differences and the spectral mismatch that exists between the PAN image and the corresponding MS image [12,29,30]. Compared to CS-based methods, MRA-based methods achieve superior spectral fidelity but the improvement of spatial resolution is limited. The characteristics of model-based methods will not be discussed as the merits and demerits of such methods vary with the specific model applied. For further knowledge, multiple studies are available for inspection [1,4,9,31,32,33,34,35]. In addition, the spectral characteristics of the PAN image is claimed to have impacts on the quality of pan-sharpened image [36].

The exploration of compressed sensing in improving pan-sharpening performance is well documented [37,38]. Compressed sensing is able to reconstruct the sparse signal (e.g., digital image) [39,40,41] in a linear combination of sparse and low-dimensional projection. Pan-sharpening methods based on compressed sensing are claimed [38,42,43] to perform better than conventional methods. For instance, the method proposed by Yang and Li [43] utilizes a slide window algorithm to obtain image patches. Then, a discrete cosine transform (DCT) dictionary and orthogonal matching pursuit (OMP) algorithm are integrated to fuse the images. Li and Yang [26] conduct pan-sharpening based on compressed sensing by randomly selecting basis atoms in a trained dictionary extracted from the PAN and MS images. Ghahremani and Ghassemian [23] propose the compressed-sensing-injection (CSI) method using a dictionary consisting only of the spatial details extracted from the PAN images by ripplet transform. Furthermore, an iterative method improving on the basis of the CSI method is proposed by Ghahremani and Ghassemian [27] to implement image pan-sharpening with the aim of preserving original spectral information. In such a method, a dictionary extracted only from the PAN images is used to reconstruct the high spatial resolution intensity images. This method is more efficient and effective for the reconstruction of high-resolution MS images compared with other studies, while only using high-resolution intensity and its sparsest coefficient. In addition, Wang et al. [44] construct the dictionary by using several sub-dictionaries composed of image patches. The patches are directly extracted from each spectral channel of an MS image. The dictionary construction takes the correlation between PAN and MS images into consideration to transform pan-sharpening into image restoration.

However, most IHS-based algorithms utilize MRA transforms to improve spectral fidelity without the aid of a compressed sensing technique, which limits the local information preservation of the pan-sharpened image [16,18,45,46]. A few methods utilize MRA tools and compressed sensing, e.g., the method based on wavelet transform and compressed sensing proposed by Cheng et al. [47]. Nevertheless, most existing studies possess intrinsic limitations in the extraction and reconstruction of spatial details using conventional MRA tools. Conventional MRA tools (e.g., discrete wavelet transform and à trous wavelet transform), have limited capability to resolve one-dimensional (1D) singularities or two-dimensional (2D), isotropic singularities. Hence, the pan-sharpened images usually suffer from Gibbs phenomena. Furthermore, curvelet transform is suggested as representing 2D singularities more efficiently. However, the scaling rule to achieve anisotropic directionality has not yet been thoroughly demonstrated.

In recent years, the shift invariant ripplet transform (RT) [48] has been proposed and extensively adopted in feature extraction. RT is able to represent 2D singularities (e.g., edges and textures) more efficiently at multiple scales and in multiple directions along a family of curves. Several studies [23,48,49] demonstrate that RT is superior to wavelet- and curvelet-based MRA transforms in terms of the effectiveness and efficiency of edge and texture extraction and reconstruction. In view of this, an IHS-based pan-sharpening method combining the high spectral fidelity of discrete ripplet transform (DRT) [49] and desirable local information preservation of compressed sensing [42,50] is proposed in this paper. Specifically, IHS transform is firstly performed on MS images to obtain the intensity component. Then, smoothing filter-based intensity modulation (SFIM) [51] is introduced to modulate spatial information contained in the intensity component without altering its spectral characteristic. The ripplet transform [23] is implemented on the intensity component and the PAN images to extract high-frequency (HF) and low-frequency (LF) sub-images. Subsequently, compressed sensing technique is applied to reconstruct the LF sub-images. Various fusion rules are adopted for the HF sub-images and LF sub-images according to their specific characteristics. The local variance algorithm [39] and local difference weighted algorithm are respectively selected as the fusion rules for HF and LF coefficients. Finally, the pan-sharpened image is obtained by inverse ripplet transform and inverse IHS transform. Experiments on WorldView-2, Pleiades and Triplesat datasets corroborate that the proposed method ensures superior spectral fidelity compared to several state-of-the-art approaches.

The reminder of the paper is structured as follows. Section 2 briefly reviews the related works. The proposed method is introduced in Section 3. The experimental results implemented on multiple RS images and discussion are provided in Section 4. Section 5 draws a brief conclusion of this paper.

## 2. Related Work

The four relevant techniques utilized in pan-sharpening are briefly reviewed here. Section 2.1 discusses the principle of the IHS-based pan-sharpening method and the cause of spectrum distortion. Section 2.2 introduces discrete ripplet transform. Sparse representation of images and the compressed sensing theory are briefly illustrated in Section 2.3.

### 2.1. IHS Transform and Spectral Distortion

The framework of IHS and IHS-like pan-sharpening methods is firstly presented in [12] and thoroughly illustrated in [52] as:

(1) $$M_{F}^{b}=M_{UP}^{b}+G_{b}\cdot \left(P_{HM}-I_{UP}\right),\;b=1,\cdots ,B$$

where $M_{F}$ is the pan-sharpened image, M denotes the original MS image, I is the intensity component separated by IHS transform, G represents the vector modulating the gains of spatial details injection, B is the number of bands in the MS image, and b denotes the bth band of the pan-sharpened or MS image. The subscript “UP” denotes the up-sampling process. In practice, the up-sampled image is produced by cubic convolution interpolation for operational convenience. It is noteworthy that this process can be implemented more properly by integrating with up-sampling, a low-pass filter and modulate transfer function (MTF) [53,54]. $P_{HM}$ is the histogram-matched PAN image. The up-sampled I component is generated by:

(2) $$I_{UP}=\overset{B}{\underset{b=1}{\sum }}w_{b}M_{UP}^{b}$$

where w is the weight vector which can be empirically set as $\frac{1}{B}$ [13] and can be experimentally chosen to weigh the spectral overlapping between PAN and MS images [24].

According to reference [12], in the inverse IHS transform for obtaining a pan-sharpened image, the input hue (H) and saturation (S) components remain unchanged, while the input (I) component is replaced by $P_{HM}$, essentially. We denote intensity variation (δ) occurs in the IHS-based pan-sharpening method which can be measured as:

(3) $$\delta =\overset{B}{\underset{b=1}{\sum }}M_{F}^{b}-M_{UP}^{b}=P_{HM}-I_{UP}$$

Due to the sensor type, local dissimilarities, and spectral mismatch between the PAN and MS images [12,29,30], δ is not equal to zero. Furthermore, the variation of I leads to a distorted S component of the pan-sharpened image. Thus, under the prerequisite of instrumentally and temporally identical pan-sharpening, spectral distortion can be remitted by decreasing δ by means of reconstructing a new intensity component that combines spectral characteristics of I and the PAN image. The proposed method is developed based on this concept. Remarkably, the maximization of image quality score index Q4 [52] is an ideal approach for distortion minimization, which is proved superior in extensive practice.

### 2.2. Discrete Ripplet Transform (DRT)

Ripplet transform [49] is proposed to address the problem that 2-D singularities along arbitrarily shaped curves cannot be resolved by conventional wavelet transform. RT generalizes the curvelet by introducing two parameters, i.e., support, c, and degree, d. The two parameters empower RT with the anisotropic capability to represent singularities along arbitrarily shaped curves.

The discretized representation of RT (i.e., DRT) is actually based on the discretization of the parameters of ripplets, which is similar to discrete curvelet transform [55]. In reference [48,49], DRT is defined at scale $a_{j}=2^{-j}$, orientation $\theta_{l}=\frac{2\Pi }{c}\cdot 2^{-\lfloor j\left(1-1/d\right)\rfloor }\cdot l$, and position ${\vec{x}}_{\vec{k}}^{j,l}=R_{\theta_{l}}^{-1}\left(c2^{-j}k_{1},2^{-j/d}k_{2}\right)$, where $\vec{k}={\left[k_{1},k_{2}\right]}^{T}$, $j,k_{1},k_{2},l\in \mathbb{Z}$, d∈ℝ, $m,n\in N,\;\mathrm{and}\;d=\frac{n}{m}$. In the frequency domain, we can represent the frequency response of the ripplet function as:

(4) $$\hat{\rho_{j}}\left(r,w\right)=c^{-\frac{1}{2}}a^{\frac{m+n}{2n}}W\left(2^{-j}\cdot r\right)V\left(c^{-1}\cdot 2^{-\lfloor j\frac{m-n}{n}\rfloor }\cdot w-l\right)$$

where the radial window W and the angular window V satisfy the following admissible conditions:

(5) $$\overset{+\infty }{\underset{j=0}{\sum }}{\left|W\left(2^{-j}\cdot r\right)\right|}^{2}=1$$

(6) $$\overset{+\infty }{\underset{i=-\infty }{\sum }}{\left|V\left(c^{-1}\cdot 2^{-\lfloor 1-d^{-1}\rfloor }\cdot w-l\right)\right|}^{2}=1$$

given c, d and j. The “wedge” corresponding to the ripplet function in the frequency domain is as follows:

(7) $$H_{j,l}\left(r,\theta \right)=\left\{2^{j}\leq \left|r\right|\leq 2^{2j},\left|\theta -2^{-\lfloor 1-d^{-1}\rfloor }\cdot l\right|\leq \frac{\pi }{2}2^{-j}\right\}$$

The DRT of an M×N image $f\left(n_{1},n_{2}\right)$ will be in the form of

(8) $$R_{j,\vec{k},l}=\overset{M-1}{\underset{n_{1}=0}{\sum }}\overset{N-1}{\underset{n_{2}=0}{\sum }}f\left(n_{1},n_{2}\right)\bar{\rho_{j,\vec{k},l}\left(n_{1},n_{2}\right)}$$

The image can be reconstructed through inverse discrete ripplet transform (IDRT):

(9) $$\tilde{f}\left(n_{1},n_{2}\right)=\underset{j}{\sum }\underset{\vec{k}}{\sum }\underset{l}{\sum }R_{j,\vec{k},l}\rho_{j,\vec{k},l}\left(n_{1},n_{2}\right)$$

### 2.3. Compressed Sensing and Sparse Representation

The basic assumption of images in sparse representation (SR) is that each original image can be represented as a linear combination of small number of atoms from a specific dictionary [42,56,57]. The main information, including local spatial features and internal structure of original images, can be concentrated in the atoms using SR. With the assumption that an original image patch s has a consistent structure, the sparse representation α of the original image patch using a specific dictionary D can be modeled as:

(10) $$\hat{\alpha }=min\;{\left\|\alpha \right\|}_{0}\;s.t.\;{\left\|D\alpha -s\right\|}_{2}^{2}=0$$

where ${\left\|\cdot \right\|}_{d=0,2}$ denotes the $L_{d}$-norm, and ${\left\|\alpha \right\|}_{0}$ is the number of non-zero components in α. Practically, directed by compressed sensing theory [42,50], we decimate a few measurements of y using decimation matrix M as:

(11) y=Ms

where $M\in {\mathbb{R}}^{n\times k}$, k is the number of atoms in the dictionary, and n≪k. Combining with the sparse representation of images and compressed sensing [27], the sparsest α ensures that image patch s can be recovered from y through the optimization process as:

(12) $$\hat{\alpha }=min\;{\left\|\alpha \right\|}_{0}\;s.t.\;{\left\|y-MD\alpha \right\|}_{2}^{2}\leq \varepsilon$$

where s=Dα, and ε is a fairly small constant that denotes the error tolerance. The optimization of Equation (12) is a Non-deterministic Polynomial (NP)-hard problem [26,27]. According to references [26,58], Equation (12) can be converted into the $L_{1}$-norm minimization problem as:

(13) $$\hat{\alpha }=min\;{\left\|\alpha \right\|}_{1}\;s.t.\;{\left\|y-MD\alpha \right\|}_{2}^{2}\leq \varepsilon$$

The basis pursuit (BP) algorithm [59] is the well-known and most commonly used optimization method to solve Equation (13). The image patches reconstruction using the BP algorithm can be represented in the standard linear formation as:

(14) $$minc^{T}\theta \;s.t.\;A\theta =b,\;\theta \geq 0$$

where $\theta =\left(\alpha^{+},\alpha^{-}\right),\;c=\left(1;1\right),A=\left(MD;\;-MD\right),\;b=y$, and $\alpha^{+}$ and $\alpha^{-}$ are the positive and negative value vectors of α, respectively. $\hat{\alpha }$ in Equation (12) is obtained by $\hat{\alpha }=\alpha^{+}-\alpha^{-}$. The sparsely reconstructed image is obtained by $\hat{s}=D\hat{\alpha }$.

## 3. Proposed Method

Based on the concept introduced in Section 2.1, the proposed method focuses on reducing spectral distortion δ by obtaining a reconstructed I component with spatial details and spectral characteristics from the PAN and MS images, respectively. We perform SFIM on the I component and the PAN image at first, and then utilize DRT to obtain HF and LF sub-images of the I component. The local variance algorithm is adopted as the fusion rule for the HF coefficient containing spatial information such as textures and edges. The LF coefficient is reconstructed using a compressed sensing technique and consequently fused according to local differences.

### 3.1. Spectral Fidelity Improvement of Intensity Component Using Sfim

In the proposed method, SFIM is performed on the PAN images and the I component to generate the new I component ($I_{SFIM}$) with modulated spatial details while spectral diversity is maintained. SFIM has two advantages which encourage its adoption for improving the spectral fidelity of $I_{SFIM}$. On one hand, the smoothing filter kernel of SFIM leads to a reduction of co-registration accuracy sensitivity. On the other hand, SFIM can be performed on individual color components. The smoothing filter kernel is an average function to obtain a local average on each pixel in a high-resolution PAN image. A smoothing filter kernel with size of s×s can be represented as:

$$\frac{1}{s}\left(\begin{array}{ccc} 1 & \cdots & 1\\ \vdots & \ddots & \vdots \\ 1 & \ldots & 1 \end{array}\right)$$

SFIM can be represented as:

(15) $$I_{SFIM}\left(x,y\right)=\frac{I\left(x,y\right)\cdot P\left(x,y\right)}{P_{Mean}\left(x,y\right)}$$

where I(x,y) is a pixel of the I component, P(x,y) is the corresponding pixel in the PAN image, and $P_{Mean}$ is the smoothed mean image of the PAN image. The size of the kernel is dependent on the ratio of resolution between the PAN and MS images, which generally equals 4 for most high resolution satellite images [51], e.g., WorldView-2, Pleiades-1A, or Triplesat (Table 1). The kernel size of the smoothing filter is not constant but changes with the experiment data. The optimization of kernel size of SFIM is experimentally discussed in Section 4.3.1.

### 3.2. Intensity Component Reconstruction Using DRT and Compressed Sensing

DRT and compressed sensing are combined to obtain a reconstructed I component ($I_{R}$) so as to adequately integrate the local spatial details and spectral information of the I component and the PAN image while minimizing the local dissimilarities and spectral mismatch between the two [12,29,30]. Specifically, DRT is advantageous to preserve the spectral features and spatial structures of source images. Compressed sensing is effective in extracting local spatial information by using image pairs [47]. In this study, two-level DRT with c=1 and d=4 is applied to obtain the HF and LF sub-images of the I component and the PAN image; note that c=1 and d=4 are shown to be sufficiently high for DRT to capture 2-D anisotropic details in high spatial resolution satellite images, such as IKONOS and QuickBird [23].

#### 3.2.1. High-Frequency Sub-Images Fusion

The HF sub-images decomposed by DRT contain sufficient spatial details of original images. The local variation of wavelet energy can represent the abundance of local information contained in the original images. The local variance of wavelet energy is utilized to fuse the high-frequency sub-images of the I component (HFI) and high-frequency sub-images of PAN images (HFP) without losing spatial details or distorting spectral characteristics [60,61,62]. Following the set-up in [61], we define the local region Q in the image with size 3×3, whose centre is (x,y). For every region Q in HFI or HFP, the principle of HF sub-images fusion rule is defined as:

(16) $$V\left(x,y\right)=\frac{1}{3\times 3}{\left(H\left(x,y\right)-\bar{H}\right)}^{2}$$

where V is the local variance value of the pixel, $\bar{H}$ is the mean value of a specific local region in HFI or HFP, and H(x,y) is the central pixel value. The fused high-frequency sub-images (HFF) can be obtained as:

(17) $$HFF\left(x,y\right)=\{\begin{array}{c} HFI\left(x,y\right),\;V_{HFI\left(x,y\right)}>V_{HFP\left(x,y\right)}\\ HFP\left(x,y\right),\;V_{HFI\left(x,y\right)}\leq V_{HFP\left(x,y\right)} \end{array}$$

where $V_{HFI\left(x,y\right)}$ and $V_{HFP\left(x,y\right)}$ are the local variance values of a specific pixel HFI(x,y) or HFP(x,y), respectively.

#### 3.2.2. Low-Frequency Sub-Images Fusion

The low-frequency sub-images of the I component (LFI) and the PAN image (LFP) decomposed by DRT contain the majority of the energy and spectral information of the I component and the PAN image, which are essential for pan-sharpening. Sparse representation and compressed sensing are introduced to get the representation of low-frequency sub-images efficiently and precisely. Predefined dictionaries are usually adopted to reconstruct images in multiple existing studies of image sparse representation and compressed sensing [43,56,63]. However, spectral characteristics of satellite images vary according to the sensor type, imaging location and imaging time [47]. Therefore, many pan-sharpening methods based on sparse representation and compressed sensing establish the dictionaries through image patches extracted from the original satellite images [23,27,30,43,44,64].

We construct the dictionary D using image patches of LFP sub-images. To combine the local information of LFI and LFP, we divide LFI and LFP into K small and partially overlapped image patches with size l×l and overlapping ratio σ using the patch-by-patch strategy. Following the set-up in the reference [27], all K patches from LFP sub-images are normalized, from which is subtracted the mean of all patches to form the dictionary D. We denote $D=\left\{d_{1},d_{2},\ldots ,d_{K}\right\}$ is the dictionary to be calculated, and the Gaussian random matrix is adopted as the decimation matrix M. Thus, each patch of LFI (i.e., $LFI^{k}$, k∈{1,… ,K}), should be reconstructed into $LFI_{R}^{k}$ according to Equation (13) as:

(18) $$\hat{\alpha_{LFI}^{k}}=min\;{\left\|\alpha^{k}\right\|}_{1}\;s.t.\;{\left\|y_{LFI}^{k}-MD\alpha^{k}\right\|}_{2}^{2}\leq \varepsilon ,\;k=1,\ldots \;,K$$

where $y_{LFI}^{k}$ is column vector lexicographically rearranged from $LFI^{k}$. According to Section 2.3, the estimated kth patch is generated as $\hat{LFI_{R}^{k}}=D\hat{\alpha_{LFI}^{k}}$ via the BP algorithm.

To ensure the fused patch is capable of integrating both the local wavelet energy and structures from LFI and LFP, the local difference weighted algorithm [62,65] is adopted to fuse $LFI_{R}^{k}$ and the corresponding kth LFP patch ($LFP^{k}$). The local difference weighted value calculated in the patch with size l×l can be represented as:

(19) $$\bar{W_{\left(x,y\right)}}=\frac{\sum_{i=x-k}^{x+k}\sum_{j=y-k}^{y+k}\left|LFI_{R}^{k}\left(i,j\right)-LFP^{k}\left(i,j\right)\right|}{l\times l}$$

where k=⌊l/2⌋, $LFI_{R}^{k}\left(i,j\right)$ and $LFP^{k}\left(i,j\right)$ denote the pixel values of a specific $LFI_{R}$ patch and corresponding $LFP^{k}$ patch, $\bar{W_{\left(x,y\right)}}$ is the local difference weighted value, and (x,y) is the central coordinate of this specific patch. The fusion rule is defined as:

(20) $$LFF_{R}^{k}\left(x,y\right)=\lambda \left[a\times LFI_{R}^{k}\left(x,y\right)+b\times LFP^{k}\left(x,y\right)\right]+\mu$$

where $LFF_{R}^{k}\left(x,\;y\right)$ is the kth fused low-frequency patch, and λ is the credibility of the pixel value λ∈(0,1]. Furthermore, *a*, *b* and μ can be defined as:

(21) a=1−b

(22) $$b=\frac{\bar{W_{\left(x,y\right)}}}{max\left[LFI_{R}^{k}\left(i,j\right)-LFP^{k}\left(i,j\right),1\leq i\leq M,1\leq j\leq N\right]}$$

(23) $$\;\mu =\left(1-\lambda \right)\times \frac{\sum_{i=1}^{l}\sum_{j=1}^{l}LFP^{k}\left(i,j\right)}{l\times l}$$

**Figure 1:**
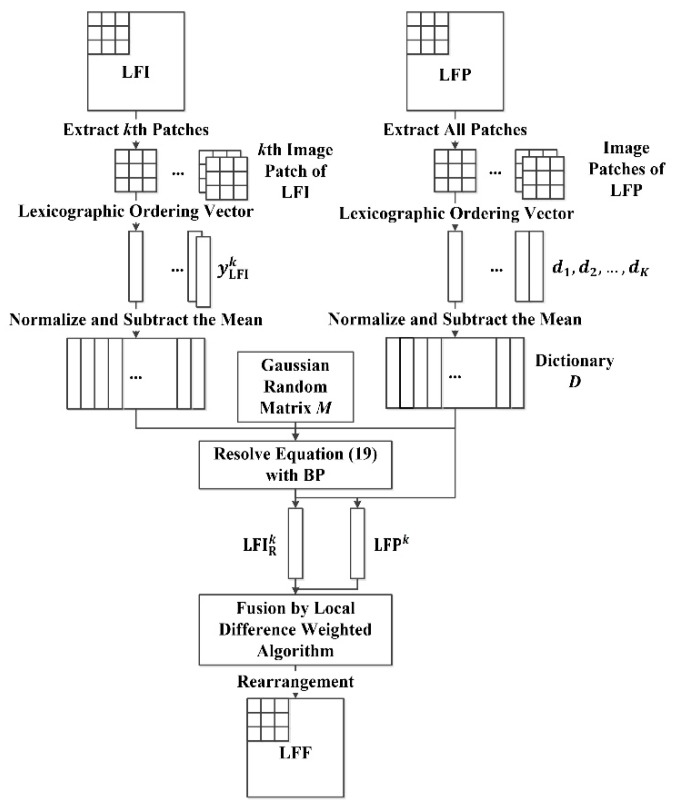
The procedure of low-frequency sub-images fusion.

The fused low-frequency patches LFF not only contain the spectral information in LFI but also preserve the characteristics of LFP, which differs from several pan-sharpening methods based on sparse representation and compressed sensing that have been recognized as excellent [23,26,47]. Subsequently, LFF are obtained by rearranging $LFF_{R}$ into image patches with the same size as LFI and LFP. The procedure of low-frequency sub-images fusion is represented by the flowchart in Figure 1.

The four main steps of low-frequency sub-images fusion can be briefly summarized as: (1) The LFI and LFP sub-images are separately divided into K small and partially overlapped image patches. All the image patches are normalized before the mean of each patch is subtracted. (2) All K patches from LFP are utilized for the dictionary composition. (3) For each patch from LFI, compressed sensing is performed by solving Equation (18) to acquire the reconstructed LFI patch. (4) The LFF patch is fused using a local difference weighted algorithm.

### 3.3. The Over-All Pan-Sharpening Method

**Figure 2:**
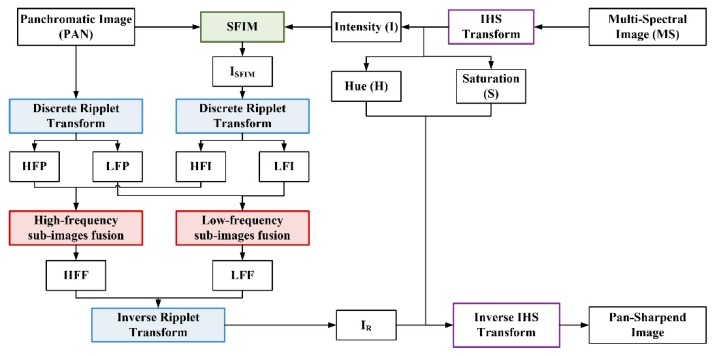
The technical flow of the proposed pan-sharpening method.

The technical flow of the proposed method is shown in Figure 2. The basic steps of the improved IHS pan-sharpening method based on ripplet transform and compressed sensing are represented as follows:

1. Data pre-processing: The MS and PAN images are co-registered precisely at first. In the meantime, the MS image is up-sampled into the same pixel size and spatial scale as PAN image using cubic convolution. This step ensures the MS and PAN images have the same pixel size and geographic coordinates.
2. IHS transform: The MS image is converted into IHS space to obtain the I component according to Equation (2). The histogram of the I component and of the PAN image are matched.
3. SFIM processing: SFIM is performed on the I component and the PAN image based on Equation (15) to obtain $I_{SFIM}$. In SFIM, the smoothing filter size should be defined experimentally.
4. DRT decomposition: The new intensity component ($I_{SFIM}$) and the PAN image are decomposed using two-level DRT with c=1 and d=4. The high frequency sub-images (HFI, HFP) and low frequency sub-images (LFI, LFP) are generated by performing DRT.
5. High-frequency sub-images fusion: HFI and HFP are fused to obtain HFF according to the local variance algorithm in Section 3.2.1.
6. Low-frequency sub-images fusion: Compressed sensing and the local weighted algorithm discussed in Section 3.2.2 is performed on LFI and LFP to obtain LFF.
7. IDRT transform: IDRT is performed on HFF and LFF to obtain the reconstructed intensity I component ($I_{R}$) following Equation (9).
8. Inverse IHS transform: By inverse IHS transform, we transform the reconstructed intensity component ($I_{R}$) and original H component and S component back into multi-band spaces to obtain the pan-sharpened image.

## 4. Results and Discussion

In this study, the proposed method, together with five state-of-the-art existing methods, is implemented on three datasets (WorldView-2, Pleiades-1A, and TripleSat) each with size 1024×1024 for comparison. The specific spatial resolution, spectral response, quantization value, and data source of the three datasets are listed in the Table 1. The datasets cover land objects such as buildings, roads, bridges, farmlands, rivers, shoreline, etc. To guarantee the generality of the experiment, we only fuse three spectral channels in the MS image: red (R), green (G), and blue (B). In fact, the proposed method on the basis of IHS transform has the potential to be extended to fuse four or more spectral bands.

The proposed method is compared with five state-of-the-art pan-sharpening methods, including adaptive IHS (AIHS) [13], wavelet transform and sparse representation (WT-SR) [66], wavelet based IHS (WT-IHS) [19], curvelet transform and independent component analysis (CT-ICA) [67], and ripplet transform based on injected details (CSI) [23]. Moreover, GS sharpening [15] is a well-known method shown to be outstanding on many satellite images for its generality and effectiveness. For fair comparison, the GS method is utilized as a standard benchmark in this study. The corresponding performances are evaluated by six quality indices. All the algorithms and indices used in experiments are implemented in MATLAB 2015b on a macOS 10.13 laptop.

In order to balance the performance and computation time of the proposed method, we empirically define the averaging filter kernel size as 5×5 when performing SFIM, and set the image patch size of 7×7 with an overlapping ratio of 15% in low-frequency sub-images fusion. In Section 4.3.1, we have a brief discussion on the size of averaging filter kernel selection in SFIM. The effects of the image patch size and overlap ratio on WorldView-2, Pleiades and Triplesat are analyzed in Section 4.3.2. To ensure the fairness of the comparison, the overlapping ratio of the CSI method is set to be 50% as recommended by [27].

### 4.1. Qualitative Comparison

**Figure 3:**
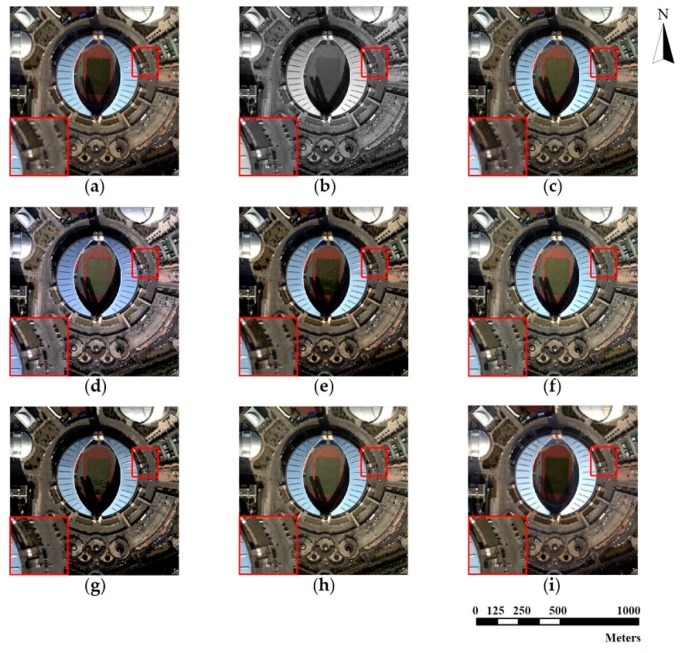
WorldView-2 original images and fusion results of different methods. (**a**) Original multispectral image (MS); (**b**) Original panchromatic image (PAN); (**c**) Gram-Schmidt [15] pan-sharpened image (GS); (**d**) Adaptive IHS [13] pan-sharpened image (AIHS); (**e**) Wavelet transform and sparse representation method [66] pan-sharpened image (WT-SR); (**f**) Wavelet-based IHS [19] pan-sharpened image (WT-IHS); (**g**) Curvelet transform and independent component analysis [67] pan-sharpened image (CT-ICA); (**h**) Ripplet transform based on injected details [23] pan-sharpened image (CSI); (**i**) pan-sharpened image by the proposed method (proposed).

**Figure 4:**
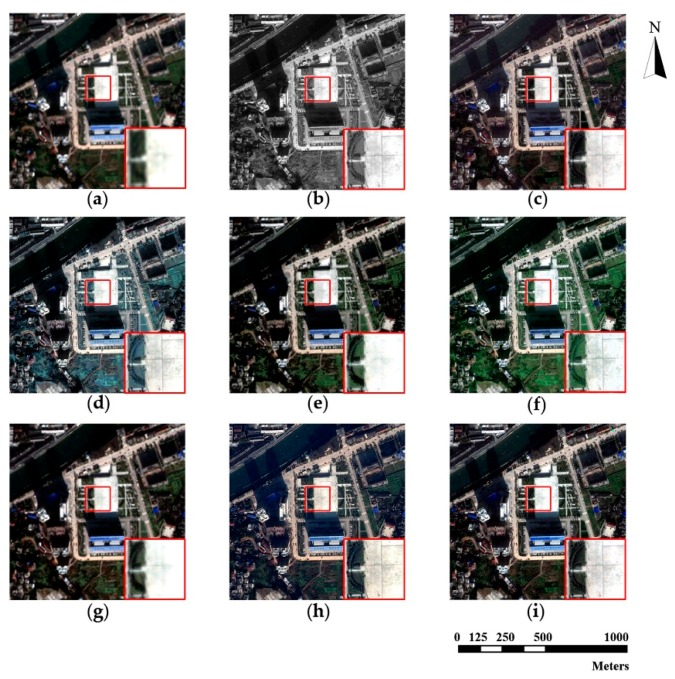
Pleiades original images and fusion results of different methods. (**a**) MS; (**b**) PAN; (**c**) GS; (**d**) AIHS; (**e**) WT-SR; (**f**) WT-IHS; (**g**) CT-ICA; (**h**) CSI; (**i**) proposed.

**Figure 5:**
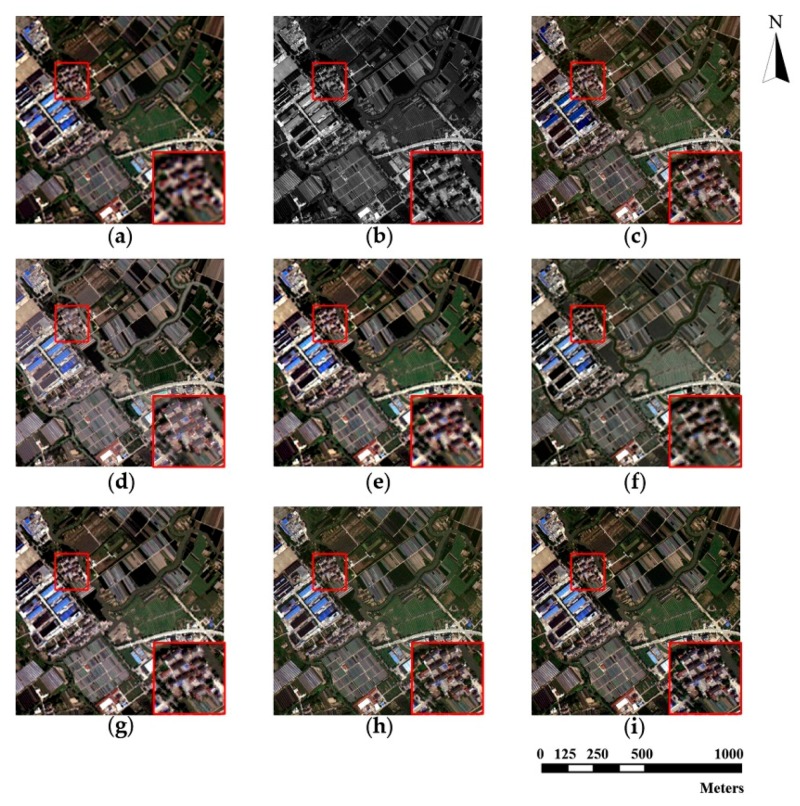
Triplesat original images and fusion results of different methods. (**a**) MS; (**b**) PAN; (**c**) GS; (**d**) AIHS; (**e**) WT-SR; (**f**) WT-IHS; (**g**) CT-ICA; (**h**) CSI; (**i**) proposed.

The source MS and PAN images from the three datasets and corresponding pan-sharpened images produced by seven individual approaches are presented in Figure 3, Figure 4 and Figure 5, respectively. The up-sampled MS images are regarded as ground truth and collaborated with GS sharpened images as benchmarks. For fairer comparisons, the zoomed images for the local area are provided at the bottom corner of each image.

The pan-sharpening results reveal that the GS method possesses ideal universality for the three experimental datasets. The GS method is able to enrich spatial information significantly with a tolerable spectral distortion. Furthermore, in general, the proposed method outperforms all the other methods (including the GS method) on the three datasets. It can be seen in Figure 3, Figure 4 and Figure 5 that the pan-sharpened image of the proposed method has the closest spectral characteristics of MS images and possesses more abundant (or approximately equivalent) spatial information than other pan-sharpened images.

From the perspective of spatial details upgrading, AIHS and WT-IHS methods perform better than WT-SR and CT-ICA. The spatial structures and textures contained in pan-sharpened images output by AIHS and WT-HIS are more visually cognizable. The AIHS method causes more obvious spectral distortion on the three datasets, which is deduced by the highest brightness of pan-sharpened images by AIHS. However, from the perspective of the preservation of spectral information, WT-SR and CT-ICA results reveal no obvious visual spectral diversity degradation. However, the zoomed images suggest these methods (especially WT-SR) perform poorly in improving spatial details. It can also be seen that the CSI method has a remarkable performance on the WorldView-2 and Triplesat datasets.

**Figure 6:**
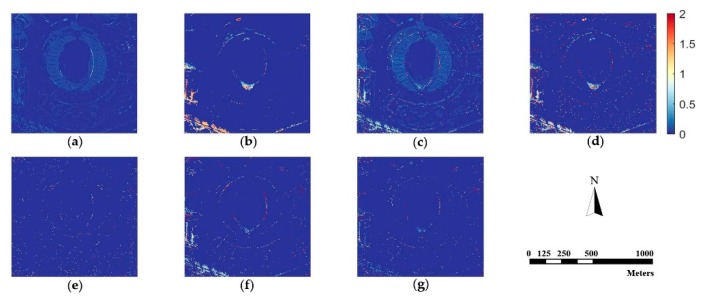
The difference image between the fused images and the referenced MS image for WorldView-2 data. (**a**) GS; (**b**) AIHS; (**c**) WT-SR; (**d**) WT-IHS; (**e**) CT-ICA; (**f**) CSI; (**g**) proposed.

**Figure 7:**
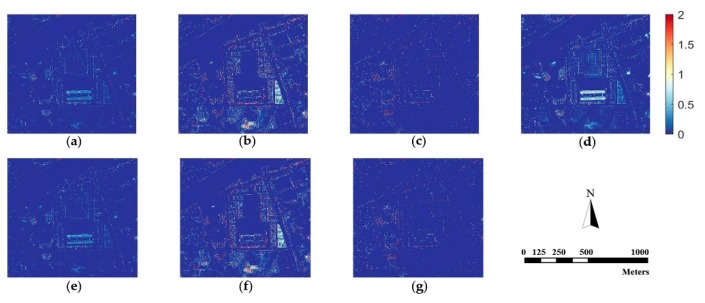
The difference image between the fused images and the referenced MS image for Pleiades data. (**a**) GS; (**b**) AIHS; (**c**) WT-SR; (**d**) WT-IHS; (**e**) CT-ICA; (**f**) CSI; (**g**) proposed.

**Figure 8:**
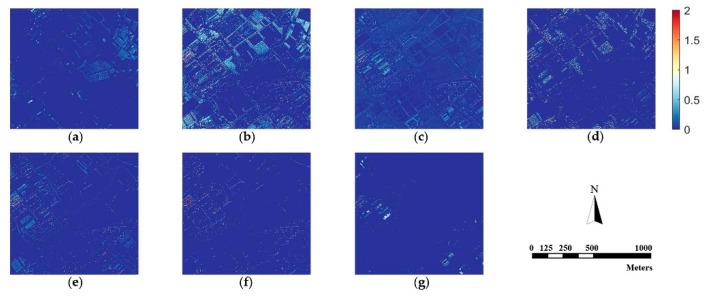
The difference image between the fused images and the referenced MS image for Triplesat data. (**a**) GS; (**b**) AIHS; (**c**) WT-SR; (**d**) WT-IHS; (**e**) CT-ICA; (**f**) CSI; (**g**) proposed.

Since human vision is not sensitive enough to perceive spectral distortion, we calculate the absolute difference values between the fusion results and the original MS image according to Equation (24) as:

(24) $$Difference\left(x,y\right)=\frac{1}{B}\overset{M}{\underset{x=1}{\sum }}\overset{N}{\underset{y=1}{\sum }}\overset{B}{\underset{b=1}{\sum }}\left|F\left(x,y,b\right)-R\left(x,y,b\right)\right|$$

where (x,y) is the coordinate of a specific pixel, B is the number of bands, M×N is the size of images, and F and R denote the pan-sharpened image and referenced MS image. Figure 6, Figure 7 and Figure 8 are difference images, which denote the absolute values of spectral information difference between each fused image and referenced MS image on every pixel for the three datasets. Blue represents finer differences and red suggests larger differences in spectral information.

Figure 6, Figure 7 and Figure 8 demonstrate that the proposed method is superior to other methods in spectral fidelity improvement. However, when implemented on the Triplesat dataset, the proposed method (Figure 8g) causes a slight spectral distortion. The difference images of the GS method are generally fine for all datasets when compared with other methods. In particular, the difference images reveal that the AIHS and WT-IHS methods lead to more serious spectral degradation, which is in accordance with visual analyses.

### 4.2. Quantitative Comparison

In quantitative comparison, correlation coefficient (CC) [10], root mean square error (RMSE) [47], the relative global synthesis error (ERGAS) [10], spectral angle mapper (SAM) [10], image quality score index (Q4) [68], and quality with no reference (QNR) [69] are adopted to evaluate the performance of individual pan-sharpening methods. Specifically, the application of CC, RMSE, ERDAS, SAM, Q4, and QNR with $D_{\lambda }$ can assess the spectral correlation between the original MS image and the pan-sharpened image. The standard deviation (STD) and QNR with $D_{s}$ reflect the richness of spatial details in the MS and pan-sharpened images. The ideal values of CC, Q4 and QNR are 1. The smaller the values for RMSE, ERGAS and SAM, the better. The quantitative results of these different methods on the three datasets are reported in Table 2, Table 3 and Table 4, respectively. For better observation, the optimal value of each quality metric is labeled in bold.

The quantitative comparison results, as represented in Table 2, Table 3 and Table 4, reveal the proposed method to be superior than other methods as it achieves the optimum value for most quality indices. Such a result is in accordance with our previous qualitative comparison. The CSI method achieves comparable values for the WorldView-2 dataset (specifically for STD of the red band and SAM) and the Triplesat dataset (specifically for CC, RMSE, STD of the blue and red bands and $D_{s}$) but fails to achieve the same overall (i.e., average) score for the WorldView-2 and Pleiades datasets. The AIHS method has relatively higher STD values on the three datasets, and even achieves the highest value of STD of the red band for the Pleiades dataset. Similar to the WT-IHS method, however, the AIHS method is also not capable of maintaining spectral information, with inferior values for CC, RMSE ERGAS, SAM and Q4. After the proposed method, CSI method performs best on the WorldView-2 and Triplesat datasets.

### 4.3. Discussion on Independence Factors and Contributions

In this section, we discuss the three independence factors of the proposed method and briefly conclude the contributions. In Section 4.3.1, the influence of smoothing filter kernel size in SFIM is discussed. The dependence of image patch size and overlapping ratio in low-frequency sub-images fusion is investigated in Section 4.3.2. The contributions are provided in Section 4.3.3.

#### 4.3.1. Discussion on Averaging Filer Kernel Size in SFIM

As mentioned in Section 3.1, the definition of averaging filer kernel size in SFIM depends on the scale ratio between PAN and MS images. The scale ratio of WorldView-2, Pleiades and Triplesat is 4 (Table 1), but the kernel size is theoretically odd [51]. Hence, 3×3 and 5×5 are the two potential optimal kernel sizes of the experiment datasets. Following the guidance in [51], we derive the CC between the pan-sharpened images with the original PAN and MS images for the three datasets. The specific results, as reported in Table 5 (where “PS” represents the pan-sharpened images), reveal 5×5 as the optimal kernel size for the study, as the sums of CC between PS with PAN and PS with MS are relatively higher than that of 3×3.

Furthermore, we increase the smoothing kernel size from 7×7 towards 13×13 to discuss the influence of kernel size in SFIM. It is noteworthy that the CCs of PS and PAN, and PS and MS become gradually identical with the increase in kernel size. Such a trend reveals that the large kernel size will excessively modulate MS images towards PAN images.

#### 4.3.2. Dependence on Image Patch Size and Overlapping Ratio

According to Section 3.2.2, image patch size l×l and the overlapping ratio σ are the only two parameters in LF sub-images fusion. The formation of dictionary D is dependent on image patch size and overlapping ratio. There is a trade-off between the completeness (i.e., number of image patches) and compactness (i.e., size of image patches) of the dictionary [70]. On one hand, a too-large image patch size accelerates computation but weakens the ability of compressed sensing to reconstruct the LF component. On the other hand, a too-small image patch size leads to high coherence of atoms in the dictionary.

We increase image patch size from 5×5 to 11×11 and the overlapping ratio from 5% to 20%. Experimental results on WorldView-2, Pleiades-1A, and Triplesat data are reported in Table 6, Table 7 and Table 8 (where “Avg” means the average value of the index of all the spectral channels), separately.

According to this statistical evidence, we can draw three conclusions:

1. The increase of the overlapping ratio σ is beneficial to performance but leads to large increases in computation time.
2. For WorldView-2, Pleiades-1A, and Triplesat data, 7×7 is the optimal image patch size.
3. In our experiments, σ=15% is the most desirable value to balance performance and computation time.

#### 4.3.3. Contributions

In general, comprehensive evaluations, both visually and by objective indices, reveal the proposed method as optimal for datasets from WorldView-2, Pleiades, and Triplesat, when compared with AIHS [13], WT-SR [66], WT-IHS [19], CT-ICA [67], CSI [23] and GS [15]. Notably, the CSI method also achieved comparable results with the exception of the Pleiades dataset. The results indicate that the proposed method achieves better spectral fidelity while yielding greater (or equivalent) spatial information improvement than the other state-of-the-art methods. Furthermore, the results demonstrate that the integration of DRT and compressed sensing can ensure the balance of spatial detail reconstruction and spectral diversity preservation.

## 5. Conclusions

In this paper, an improved IHS-based pan-sharpening method using ripplet transform and compressed sensing is proposed, which overcomes the spectral distortion that occurs in the original IHS method. Firstly, SFIM is performed on the PAN image and the I component separated from MS image to improve spectral fidelity. The proposed method adopts different fusion rules according to individual characteristics of the HF and LF coefficients obtained by DRT. The fusion rule based on local variance of wavelet energy is utilized for HF sub-images. For LF information, we use a compressed sensing technique to maintain the local structures and spectral information. The LF coefficients of the I component and the PAN image are divided into small patches and a dictionary is constructed using patches of low-frequency components of PAN. The reconstructed low-frequency components of the I component and the PAN image are generated using the BP algorithm, and the local difference weighted algorithm is used to generate new LF components. For the combination of IHS transform, DRT and compressed sensing, the proposed algorithm can produce pan-sharpened images with both high spatial and high spectral resolution efficiently and effectively.

The performance of the proposed method and five state-of-the-art methods is comprehensively evaluated using WorldView-2, Pleiades-1A and Triplesat datasets. The specific evaluation indices include CC, RMSE, STD, ERGAS, SAM, Q4 and QNR. The experimental results demonstrate that the proposed method performs better than other fusion methods and overcomes spectral distortion while improving spatial resolution significantly. In future work, more effort should be dedicated to improving the efficiency of the proposed method, as the BP algorithm is time-consuming and can thus be potentially replaced by other reconstruction algorithms. In addition, the practicability of the algorithm images captured by other remote sensors has yet to be demonstrated.

## Figures and Tables

**Table 1:** Characteristics of three datasets used in this study.

Features	WorldView-2			Pleiades-1A		Triplesat	
	PAN	MS		PAN	MS	PAN	MS
**Spatial Resolution**	0.46 m	1.84 m		0.5 m	2 m	0.8 m	3.2 m
**Spectral Response**	450–800 nm	Costal (C): 400–450 nm	Red (R): 630–690 nm	470–830 nm	Blue (B): 430–550 nm	450–650 nm	Blue (B): 440–510 nm
		Blue (B): 450–510 nm	Red Edge (RE): 705–745 nm		Green (G): 500–620 nm		Green (G): 510–590 nm
		Green (G): 510–580 nm	Near IR-1 (NIR-1): 770–895 nm		Red (R): 590–710 nm		Red (R): 600–670 nm
		Yellow (Y): 585–625 nm	Near IR-2 (NIR-2): 860–900 nm		Near IR (NIR): 740–940 nm		Near IR (NIR): 760–910 nm
**Quantization Value**	11 Bits			12 Bits or 16 Bits		10 Bits	
**Imaging Location**	Huang Long Stadium in Hangzhou, China			Xuanen county government in Enshi, China		Qingpu district in Shanghai, China	
**Imaging Time**	20 December 2009			13 April 2015		18 September 2017	

**Table 2:** Evaluation indices of fusion results for WorldView-2 data.

Index		GS	Method					
			AIHS	WT-SR	WT-IHS	CT-ICA	CSI	Proposed
**Correlation coefficient (CC)**	R	0.945	0.896	0.912	0.910	0.938	0.941	**0.952**
	G	0.936	0.879	0.934	0.915	0.924	0.953	**0.957**
	B	0.940	0.925	0.927	0.912	0.947	0.948	**0.949**
	Average	0.941	0.900	0.924	0.912	0.936	0.947	**0.953**
**Root mean square error (RMSE)**	R	15.02	16.96	15.67	16.24	**14.68**	15.17	14.91
	G	14.86	16.85	15.94	16.36	14.96	13.82	**13.64**
	B	14.07	17.23	16.07	15.91	15.24	13.94	**13.65**
	Average	14.65	17.05	15.89	16.17	14.96	14.31	**14.07**
**Standard deviation (STD)**	R	63.61	59.65	58.34	63.28	62.34	**66.24**	65.27
	G	65.09	61.23	57.97	**67.95**	57.68	64.16	65.33
	B	62.82	57.29	58.26	61.27	58.29	63.90	**65.28**
	Average	63.84	59.39	58.19	64.17	59.43	64.77	**65.29**
**The relative global synthesis error (ERGAS)**		2.873	3.681	3.487	3.335	3.015	2.974	**2.762**
**Spectral angle mapper (SAM)**		5.096	7.620	8.673	7.241	6.292	**3.245**	3.271
**Image quality score index (Q4)**		0.831	0.589	0.534	0.591	0.628	0.896	**0.913**
**Quality with no reference (QNR)**	$D_{s}$	0.129	0.145	0.132	0.152	0.137	0.125	**0.121**
	$D_{\lambda }$	0.143	0.186	0.174	0.163	0.151	0.134	**0.132**
	Average	0.808	0.669	0.781	0.748	0.795	0.818	**0.854**

**Table 3:** Evaluation indices of fusion results for Pleiades data.

Index		GS	Method					
			AIHS	WT-SR	WT-IHS	CT-ICA	CSI	proposed
CC	R	0.833	0.675	0.806	0.713	0.867	0.821	**0.875**
	G	0.802	0.623	0.814	0.756	0.859	0.834	**0.861**
	B	0.799	0.694	0.795	0.697	**0.874**	0.806	0.868
	Average	0.811	0.664	0.805	0.722	0.867	0.820	**0.868**
RMSE	R	10.14	13.68	10.29	12.40	9.799	10.28	**9.014**
	G	9.996	13.55	10.37	12.52	9.368	9.867	**9.257**
	B	10.11	13.27	11.15	12.70	9.257	10.02	**9.119**
	Average	9.773	13.50	10.60	12.54	9.475	10.05	**9.130**
STD	R	53.02	**56.56**	47.92	51.37	52.51	55.69	55.24
	G	53.97	52.46	46.58	52.17	53.11	54.72	**55.67**
	B	54.12	53.65	47.14	52.64	52.89	55.02	**55.94**
	Average	53.70	54.16	47.22	52.00	52.84	55.15	**55.62**
ERGAS		1.709	2.689	2.054	2.337	1.680	1.806	**1.547**
SAM		1.643	2.081	1.854	1.922	1.725	1.734	**1.549**
Q4		0.878	0.657	0.762	0.715	0.865	0.890	**0.894**
QNR	$D_{s}$	0.119	0.264	0.181	0.254	0.213	0.157	**0.119**
	$D_{\lambda }$	0.102	0.118	0.097	0.154	0.105	0.094	**0.086**
	Average	0.810	0.675	0.834	0.760	0.798	0.825	**0.874**

**Table 4:** Evaluation indices of fusion results for Triplesat data.

Index		GS	Method					
			AIHS	WT-SR	WT-IHS	CT-ICA	CSI	proposed
CC	R	0.905	0.757	0.852	0.884	0.939	0.961	**0.975**
	G	0.917	0.778	0.867	0.904	0.928	0.963	**0.982**
	B	0.893	0.802	0.840	0.898	0.922	**0.975**	0.966
	Average	0.905	0.779	0.853	0.895	0.930	0.966	**0.974**
RMSE	R	19.83	30.81	25.42	26.73	22.40	12.42	**10.08**
	G	16.77	29.96	24.36	28.39	22.33	12.18	**9.422**
	B	13.92	30.46	25.05	27.57	20.50	**10.75**	11.60
	Average	16.74	30.41	24.94	27.56	21.74	11.78	**10.37**
STD	R	59.80	60.06	47.56	57.01	51.66	62.17	**63.52**
	G	61.07	60.61	48.73	57.79	51.86	**62.62**	62.07
	B	60.58	59.59	47.34	56.50	50.14	**62.37**	62.17
	Average	60.48	60.09	47.88	57.10	51.13	62.39	**62.59**
ERGAS		1.402	3.925	1.875	2.613	1.534	1.394	**1.360**
SAM		1.996	5.341	3.522	4.898	2.847	2.002	**1.987**
Q4		0.810	0.675	0.806	0.761	0.861	0.925	**0.979**
QNR	$D_{s}$	0.177	0.329	0.168	0.214	0.175	**0.137**	0.149
	$D_{\lambda }$	0.138	0.205	0.177	0.198	0.154	0.116	**0.103**
	Average	0.810	0.668	0.715	0.694	0.767	0.832	**0.869**

**Table 5:** Impact of smoothing filer kernel size in SFIM on three datasets.

Dataset	CC	Kernel Size					
		3 × 3	5 × 5	7 × 7	9 × 9	11 × 11	13 × 13
WorldView-2	PS and PAN	0.738	0.765	0.776	0.797	0.831	0.841
	PS and MS	0.969	0.958	0.935	0.902	0.881	0.867
	Sum	1.707	1.723	1.721	1.699	1.712	1.708
Pleiades	PS and PAN	0.715	0.731	0.742	0.757	0.772	0.791
	PS and MS	0.879	0.868	0.847	0.833	0.825	0.802
	Sum	1.594	1.599	1.589	1.590	1.577	1.593
Triplesat	PS and PAN	0.732	0.788	0.805	0.835	0.842	0.846
	PS and MS	0.981	0.974	0.955	0.921	0.913	0.902
	Sum	1.713	1.762	1.760	1.756	1.755	1.746

**Table 6:** Impact of image patch size and overlapping ratio on WorldView-2 image.

Image Patch Size	Overlapping Ratio	CC (Avg)	RMSE (Avg)	STD (Avg)	ERGAS	SAM	Q4	QNR (Avg)	Time (s)
5 × 5	5%	0.887	19.02	55.21	3.330	4.315	0.804	0.751	40.91
5 × 5	10%	0.906	18.27	57.93	3.205	4.298	0.827	0.766	56.43
5 × 5	15%	0.921	17.49	59.66	3.119	4.102	0.840	0.794	82.72
5 × 5	20%	0.934	17.33	60.57	3.012	3.926	0.856	0.801	107.5
7 × 7	5%	0.949	16.21	63.01	2.887	3.614	0.891	0.835	61.25
7 × 7	10%	0.951	14.95	64.67	2.824	3.359	0.907	0.847	85.60
7 × 7	15%	0.953	14.07	65.29	2.762	3.271	0.913	0.854	119.7
7 × 7	20%	0.954	13.65	65.33	2.637	3.153	0.918	0.862	162.1
9 × 9	5%	0.924	14.73	63.29	2.835	3.825	0.882	0.826	72.71
9 × 9	10%	0.930	14.65	63.37	2.829	3.814	0.897	0.833	105.8
9 × 9	15%	0.935	14.58	63.45	2.820	3.802	0.903	0.839	156.1
9 × 9	20%	0.937	14.46	63.58	2.813	3.796	0.909	0.841	182.9
11 × 11	5%	0.918	15.32	62.09	2.964	3.967	0.873	0.811	86.50
11 × 11	10%	0.924	15.24	62.17	2.947	3.956	0.879	0.817	117.1
11 × 11	15%	0.931	15.13	62.22	2.939	3.943	0.885	0.824	165.6
11 × 11	20%	0.935	15.09	62.36	2.932	3.937	0.891	0.829	196.4

**Table 7:** Impact of image patch size and overlapping ratio on Pleiades-1A image.

Image Patch Size	Overlapping Ratio	CC (Avg)	RMSE (Avg)	STD (Avg)	ERGAS	SAM	Q4	QNR (Avg)	Time (s)
5 × 5	5%	0.788	10.267	44.328	2.339	2.357	0.786	0.781	24.26
5 × 5	10%	0.795	9.901	46.583	2.328	2.340	0.801	0.793	59.67
5 × 5	15%	0.813	9.825	48.772	2.224	2.214	0.815	0.805	80.41
5 × 5	20%	0.821	9.658	49.935	2.107	2.098	0.834	0.829	95.93
7 × 7	5%	0.837	9.246	53.839	1.715	1.772	0.863	0.847	49.47
7 × 7	10%	0.854	9.224	54.348	1.606	1.618	0.879	0.861	80.65
7 × 7	15%	0.868	9.130	55.617	1.547	1.549	0.894	0.874	124.3
7 × 7	20%	0.882	9.067	56.811	1.435	1.421	0.912	0.886	165.7
9 × 9	5%	0.811	9.425	51.267	1.809	1.795	0.851	0.835	50.66
9 × 9	10%	0.820	9.319	51.983	1.775	1.682	0.860	0.842	61.42
9 × 9	15%	0.829	9.221	52.617	1.661	1.607	0.873	0.856	98.71
9 × 9	20%	0.836	9.178	53.209	1.587	1.534	0.889	0.868	174.9
11 × 11	5%	0.805	9.729	50.664	1.924	1.895	0.847	0.822	72.83
11 × 11	10%	0.814	9.633	51.709	1.873	1.862	0.851	0.829	103.6
11 × 11	15%	0.827	9.521	53.105	1.705	1.734	0.864	0.838	138.7
11 × 11	20%	0.833	9.413	54.018	1.582	1.607	0.877	0.845	190.1

**Table 8:** Impact of image patch size and overlapping ratio on Triplesat image.

Image Patch Size	Overlapping Ratio	CC (Avg)	RMSE (Avg)	STD (Avg)	ERGAS	SAM	Q4	QNR (Avg)	Time (s)
5 × 5	5%	0.883	11.304	51.178	2.016	3.357	0.917	0.798	35.34
5 × 5	10%	0.898	11.295	52.591	1.912	3.102	0.921	0.803	62.40
5 × 5	15%	0.907	11.187	53.834	1.887	2.945	0.925	0.811	105.4
5 × 5	20%	0.924	11.062	54.367	1.654	2.798	0.934	0.826	162.7
7 × 7	5%	0.951	10.563	60.498	1.456	2.223	0.955	0.847	84.64
7 × 7	10%	0.962	10.445	62.067	1.398	2.148	0.968	0.853	125.3
7 × 7	15%	0.974	10.368	62.586	1.360	1.987	0.979	0.869	141.5
7 × 7	20%	0.989	10.017	63.054	1.349	1.905	0.982	0.874	188.4
9 × 9	5%	0.941	10.664	55.392	1.489	2.482	0.940	0.841	94.51
9 × 9	10%	0.952	10.532	56.018	1.447	2.405	0.954	0.849	110.9
9 × 9	15%	0.966	10.467	56.981	1.394	2.349	0.963	0.855	145.2
9 × 9	20%	0.975	10.395	57.765	1.251	2.297	0.977	0.863	191.6
11 × 11	5%	0.933	10.894	56.215	1.664	2.664	0.933	0.832	86.32
11 × 11	10%	0.941	10.752	57.541	1.598	2.615	0.941	0.839	119.7
11 × 11	15%	0.957	10.663	58.165	1.435	2.533	0.956	0.847	158.5
11 × 11	20%	0.969-	10.597	59.085	1.357	2.498	0.968	0.851	200.2
